# Kneading‐Inspired Versatile Design for Biomimetic Skins with a Wide Scope of Customizable Features

**DOI:** 10.1002/advs.202200108

**Published:** 2022-03-22

**Authors:** Jiahui Huang, Peiyi Wu

**Affiliations:** ^1^ State Key Laboratory of Molecular Engineering of Polymers Department of Macromolecular Science and Laboratory of Advanced Materials Fudan University Shanghai 200433 China; ^2^ State Key Laboratory for Modification of Chemical Fibers and Polymer Materials College of Chemistry Chemical Engineering and Biotechnology Center for Advanced Low‐Dimension Materials Donghua University Shanghai 201620 China

**Keywords:** biomimetic skins, customized features, hydrogels, skin‐like materials, viscoelastic hydrogels

## Abstract

Biomimetic skins featuring customizable functions and human tissue‐compatible mechanical properties have garnered tremendous interest for potential applications in human–machine interfaces, flexible wearable devices, and soft robotics. However, most existing skin‐like materials require complex molecular design or multistep functionalization to achieve various functionalities that match or even surpass the performance of human skin. Thus, simultaneously minimizing production costs and achieving customizable features are still highly desirable yet challenging. Herein, inspired by a well‐known kneading technique that renders a homogeneous mixture of all the ingredients, a versatile method involving two steps of kneading and resting is employed to prepare biomimetic skins with a wide scope of customizable features. Commonly used one‐dimensional (1D), two‐dimensional (2D), three‐dimensional (3D) nanofillers and even solvents are demonstrated to be homogeneously dispersed in the viscoelastic hydrogel matrices by hand kneading, which not only contributes to improved mechanical properties and new functionalities, but also makes full use of raw materials without waste. Furthermore, similar to the combination of “condiments” in kneading dough, the flexible integration of functional fillers offers exciting and versatile platforms for the design of biomimetic skins with tunable application‐specific properties, such as mechanical compliance, sensory capabilities, freezing resistance, 3D printability, fluorescence tunability, etc.

## Graphical abstract

Inspired by a well‐known kneading technique, a facile yet versatile kneading method for engineering biomimetic skins with a wide spectrum of customizable features is reported. Typical nanomaterials and even solvents can be homogeneously dispersed in the viscoelastic hydrogel matrices by hand kneading, which not only contributes to improved mechanical properties and new functionalities, but also makes full use of raw materials.

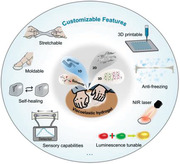

## 1 Introduction

Human skin, which combines a broad spectrum of mechanical properties and multiple sensory capabilities toward different stimuli, plays an essential role in mediating our interactions with the world.^[^ ^1^, ^2^, ^3^ ^]^ These integrated traits are highly intelligent and have inspired the development of conductive materials as biomimetic counterparts (i.e., electronic or ionic skin) for a broad scope of applications, including sensors,^[^ ^4^, ^5^ ^]^ human–machine interfaces,^[^ ^6^, ^7^ ^]^ flexible wearable devices,^[^ ^8^, ^9^ ^]^ and soft robotics.^[^ ^10^, ^11^ ^]^ Particularly, due to the great similarity in physiological and mechanical properties to biological materials and soft tissues, conductive hydrogels have flourished as desirable candidates for artificial skin‐like sensors.^[^ ^12^ ^]^ But at the same time, higher requirements, even customized features, are placed on these biomimetic skin‐like materials to meet the needs under complex working conditions. For example, they ought to be stretchable and compliant to reconfigure themselves to adapt to irregular and dynamic surfaces.^[^ ^13^, ^14^ ^]^ Besides, to enhance their lifetime, they need to have the ability to self‐repair when subject to damage and prevent dehydration through a non‐volatile design or an epidermal layer.^[^ ^15^, ^16^ ^]^ Moreover, for future large‐scale applications, recyclability, easy integration, and processability are also highly desired features.^[^ ^17^, ^18^ ^]^

To satisfy these sophisticated requirements, two main feasible material design strategies have been developed for the purpose of biomimetic uses. One is molecular design by fine‐tuning dynamic intermolecular interactions^[^ ^1^, ^19^, ^20^ ^]^ (e.g., hydrogen bonding, electrostatic interactions, metal‐ligand coordination, and *π*–*π* interactions) or optimizing molecular structures (e.g., adding dopants to partially soften polymer chains^[^ ^21^ ^]^). In contrast, the other attractive method is to engineer composites in which conductive components, such as electronically conductive fillers and ionic electrolytes, are embedded in the polymer matrix.^[^ ^22^, ^23^, ^24^, ^25^ ^]^ This “add‐on” approach is conductive to yielding desired functionalities derived from one or more multifunctional fillers.^[^ ^26^ ^]^ The key is to manage the compatibility between conductive paths and the insulating networks to maximize the utilization of multifunctional fillers in nanocomposite applications. Despite remarkable achievements, conventional synthesis methods for most conductive hydrogels always suffer from tedious and rigorous manufacturing processes. For example, in situ polymerization of monomers in the presence of conductive components often involves toxic monomers/crosslinkers/initiators and even an oxygen‐free environment.^[^ ^27^, ^28^ ^]^ The treatment of heat or ultraviolet and multistep functionalization also makes the preparation much time‐ and energy‐consuming, which cannot meet the need for further mass production. Moreover, once formed, it is difficult to modulate and edit their properties by changing their composition. Therefore, minimizing material waste and production costs as well as addressing the customization issues for biomimetic skins is still highly desirable yet challenging.

Inspired by kneading technique, an important and household step in the cooked wheaten food culture, which enables the homogeneous mixing of all the ingredients (wheat flour and water) either by hand or machine. The input of mechanical energy leads to new interactions between gluten proteins through disulfide bonds and promotes the development of gluten networks, resulting in a viscoelastic dough.^[^ ^29^, ^30^ ^]^ And interestingly, the subsequent adjustment of the flavor of the dough by adding additives (i.e., condiments) can also be realized by kneading and resting, in which the viscoelastic dough is utilized as a carrier. This leads to the idea that biomimetic skin‐like materials are expected to display customizable features according to the selection of “condiment” and be homogenized by kneading.

In this work, we demonstrate a facile yet versatile kneading method for engineering biomimetic skins with a wide spectrum of customizable features. With a kind of viscoelastic hydrogel as the carrier, a variety of functional fillers (i.e., commonly used one‐dimensional,1D, two‐dimensional, 2D, three‐dimensional, 3D nanofillers and even solvents) have been successfully incorporated and homogeneously dispersed in the hydrogel matrix by hand kneading, with a raw material utilization close to 100%. This is attributed to the fact that kneading facilitates homogeneous mixing of all the ingredients, and the subsequent resting is conductive to autonomous regulation and optimization of the network structure. The flexible selection and integration of diverse fillers endow resultant skin‐like materials with tunable application‐specific properties. Unlike most conductive hydrogels requiring complex molecular design or multistep functionalization, our kneading method provides a powerful versatile platform for constructing integrated functional products.

## 2 Results and Discussion

**Figure 1:**
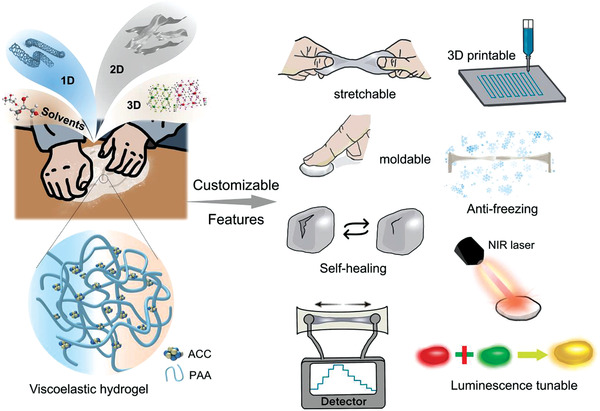
Schematics of the kneading method and the versatile design for biomimetic skins with a wide scope of customizable features.

This kneading method consists of two steps including kneading and resting, as in the dough kneading technique. Generally, kneading allows the initial homogenization and hydration of the ingredients under mechanical energy for the development of the gluten network.^[^ ^30^ ^]^ With viscoelastic dough as the carrier, it can also accommodate a variety of additives added later while retaining the viscoelastic characteristics of dough. Nevertheless, during the kneading process, air bubbles would be introduced either intentionally or accidentally.^[^ ^29^ ^]^ Besides, the undesired residual stress caused by the entanglement of protein macromolecules under external force would lead to the deterioration of the mechanical properties of the dough. As such, resting, a subsequent step to kneading, enables autonomous regulation of the network structure. Similar to the dough, hydrogels are generally recognized as viscoelastic materials, i.e., their mechanical properties demonstrate a combination of viscosity and elasticity.^[^ ^31^, ^32^ ^]^ As proof‐of‐concept demonstration of our kneading method, a dough‐like viscoelastic hydrogel (VEH) with physically cross‐linked networks composed of poly(acrylic acid) (PAA) and amorphous calcium carbonate (ACC) nanoparticles^[^ ^33^ ^]^ is selected as a model system in this work. The solid content of the fully swollen ACC/PAA hydrogel was estimated to be 40–46 wt%. The viscoelastic nature of hydrogel plays an important role in this manufacturing process, because the plasticity of the matrix facilitates the infiltration of nanomaterials and the mechanical adaptability under external force, while the elasticity renders restoration of the composite upon removing external force. As illustrated in **Figure** **1**, the commonly used 1D (e.g., carbon nanotubes, CNT), 2D (e.g., MXene and graphene oxide, GO), 3D nanofillers (e.g., metal‐organic frameworks, MOF) even solvents (e.g., ethylene glycol and glycerol) can be homogeneously dispersed in the viscoelastic hydrogel matrix by hand kneading, either alone or in combination form. These tunable and flexible “condiments” can bring customizable features and mechanical versatility to resultant composite conductive hydrogels to cope with complex requirements, such as stretchability, mechanical compliance, sensory capabilities, freezing resistance, 3D printability, photothermal responsiveness, fluorescence tunability, etc. Notably, this kneading method allows a nearly 100% utilization of raw materials.

**Figure 2:**
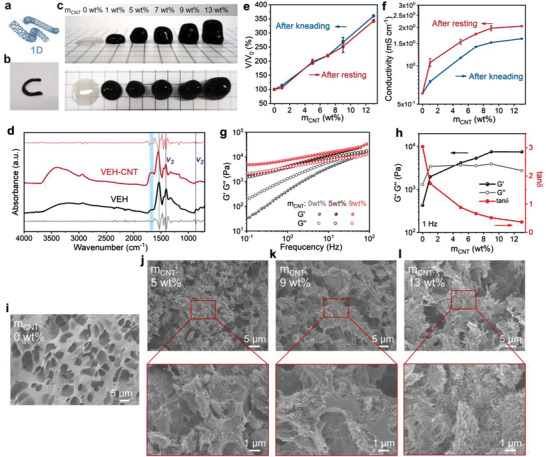
a) Structure diagram of CNTs. b) Photograph of VEH‐CNT with CNT content of 5 wt%. c) Photographs of the appearance of VEH‐CNT with different CNT contents. The side of each grid is 1 cm. d) FTIR spectra of VEH and VEH‐CNT. e) Volume changes of VEH‐CNT kneaded with different contents of CNT. f) Conductivity changes of VEH‐CNT kneaded with different contents of CNT. g) Frequency dependencies of the storage (G′) and loss (G″) moduli of VEH‐CNT with varying CNT contents. h) G′ and G″ of VEH‐CNT at the frequency of 1 Hz and corresponding loss tangent tan *δ* (G″/G′) calculations under 0.1% strain. i) SEM image of pristine VEH. SEM images of VEH‐CNT with CNT content of j) 5 wt%, k) 9 wt%, and l) 13 wt%.

The typical example of 1D nanofillers incorporated into the viscoelastic hydrogel by kneading is provided by CNTs, which contribute to substantially enhanced mechanical and electrical performance. The hydrophilic CNT paste used in our work is composed of multiwall tubes with the diameter of ≈20 nm, along with a negative‐charged surface arising from the oxygen‐containing functional groups (**Figure** **2**a and Figure S1, Supporting Information). As expected, after kneading for several minutes, the resultant viscoelastic hydrogel/CNT composite (VEH‐CNT) exhibits a uniform color appearance and inherits the plasticity of the polymer matrix, which can be remolded into different shapes, such as the letter C (Figure 2b). Interestingly, with the help of an automatic household dough maker, large‐sized VEH‐CNT can also be prepared readily with similar appearance and malleability as hand‐kneaded ones (Figure S2, Supporting Information), verifying the feasibility of further mass production.

To gain insight into the kneading process, a series of VEH‐CNTs were prepared with CNT paste mass ratios ranging from 0 to 13 wt%. As depicted in Figure 2c, their volumes show a distinct increase with the addition of CNTs after kneading and a slight decrease after resting (Figure 2e). Since in the case of hand kneading, the CNTs agglomerates are defibrated and dispersed evenly in the viscoelastic hydrogel matrix, and are connected through the interfacial phase to form a continuous structure of CNTs within the polymer. Fourier transform infrared spectra (FTIR) confirm the interfacial interaction between CNTs and viscoelastic hydrogel (Figure 2d), which is beneficial for preventing aggregation during processing.^[^ ^34^ ^]^ In the pristine VEH, the peak at 1531 cm^–1^ (*v*(COO^–^)) reveals the deprotonation of most of the carboxyl groups of PAA, suggesting the chelation between COO^–^ and Ca^2+^.^[^ ^35^ ^]^ The rise of the C═O stretching band (around 1700 cm^–1^) in the VEH‐CNT comes from the introduced CNT, a potential hydrogen‐bond acceptor, which indicates the existence of hydrogen bonds between CNTs and a small amount of unionized carboxyl groups in PAA (hydrogen‐bond donor).^[^ ^36^ ^]^ The two vibrational bands from ACC, *v_2_* (860 cm^–1^) and *v_3_* (1406 cm^–1^), appear in both VEH and VEH‐CNT, confirming the structural integrity after kneading, i.e., without leading to the crystallization of ACC. Otherwise *v*_1_ *‐v*_4_ bands will locate at very different wavenumbers for the crystalline CaCO_3_ phases, e.g., calcite (874, 713 cm^–1^), vaterite (876, 744 cm^–1^), and aragonite (854, 712 cm^–1^).^[^ ^37^ ^]^ Besides, the electrical conductivity of VEH‐CNTs also follows an ascending trend with the increase of CNTs loading, which is due to the gradual formation of a continuous conductive filler network. As CNT increases, spacings between adjacent CNTs gradually decrease, thus generating more hopping sites for electron transfer.^[^ ^34^ ^]^ And interestingly, resting for 2 h can result in improved electrical conductivity of VEH‐CNTs (Figure 2f and Figure S3, Supporting Information). For example, the VEH‐CNT with CNT content of 9 wt% exhibits a conductivity of 1.5 mS cm^–1^ after kneading, and reaches 2.0 mS cm^–1^ after resting. A possible explanation is that resting helps to eliminate air bubbles and remove the residual stress generated during the kneading process, which enables the relaxation and reconfiguration of polymer chains, while optimizing the conductive pathways constructed by CNTs. Such behavior could also account for the slight decrease in the volume of VEH‐CNT after resting.

Also, the addition of CNTs to the viscoelastic hydrogel increases its stiffness, as evidenced by dynamic rheological measurements. Due to the physically cross‐linked nature, the viscoelasticity of VEH displays a significant frequency dependence (Figure 2g). Its loss modulus (G″) is slightly higher than the storage modulus (G′), indicative of a slightly dominant role of viscosity. When the content of kneaded CNTs is increased, G′ significantly increases and the resulting VEH‐CNT possesses predominantly elastic feature (G′ > G″) as the CNTs content reaches 5 wt%, while remaining frequency‐dependent (Figure S4, Supporting Information). The loss tangent tan *δ*, defined as the ratio of G″ to G′, is applied to quantify the relative contribution of the viscous and elastic components to the material, with values less than 1 having a higher elastic contribution, and vice versa.^[^ ^38^ ^]^ The tan *δ* value of VEH‐CNT collected at 1 Hz increases monotonically with the content of CNTs, and is less than 1 (0.88) as the CNTs content reaches 5 wt% (Figure 2h), which highlights the elastic contribution from the kneaded CNTs. Scanning electron microscopy (SEM) images further offer microscopic insight. The Pristine VEH exhibits the interconnected porous architecture (Figure 2i), typical for hydrogels. As more CNTs are kneaded, SEM images reveal that the backbones of polymer chains and porous structure are not affected, accompanied by a dense, and uniform network formed by the arrangement of CNTs. Consequently, it is considered that the VEH is highly reinforced in both electrical and mechanical properties by the continuous network built by kneaded CNTs.

**Figure 3:**
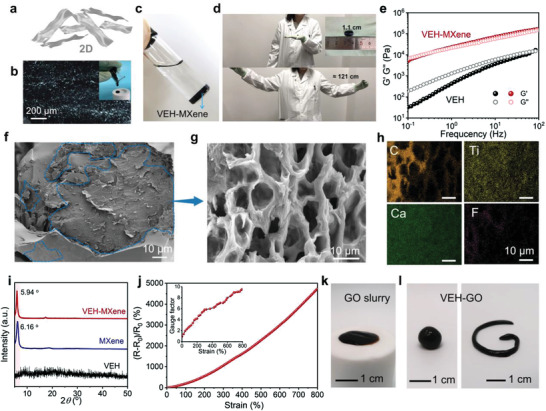
a) Schematic diagram of 2D materials with large aspect ratio. b) Polarized optical microscope (POM) image of MXene slurry, inset is its optical photograph. c) Photograph of VEH‐MXene after being stored in water for 24 h. d) Photographs showing the ultra‐stretchability of VEH‐MXene. e) G′ and G″ of VEH‐MXene and VEH. SEM images of VEH‐MXene f) just after mixing and g) after kneading and resting. h) Elemental mapping analysis of VEH‐MXene from (g). i) XRD patterns of VEH‐MXene, MXene, and VEH, respectively. j) Relative resistive signals‐strain curves and gauge factors of VEH‐MXene based sensor. k) Photographs of GO slurry. l) Photographs demonstrating the malleability of VEH‐GO.

Moreover, the extraordinarily large aspect ratio and unique properties of 2D materials rank them as superior reinforcers and modifiers for the polymer‐matrix nanocomposites. Here, we choose two popular 2D materials, MXene (Ti_3_C_2_T_x_) and GO, as proof‐of‐concept demonstration for our kneading method (**Figure** **3**a).

MXene has been well known for its outstanding electrical conductivity and excellent solution‐processing capabilities.^[^ ^39^, ^40^, ^41^ ^]^ As shown in Figure 3b, the MXene slurry (≈40 mg mL^−1^) comprised of large and high‐quality MXene flakes (Figure S5, Supporting Information) is prepared according to our previous work.^[^ ^42^ ^]^ It shows a lyotropic liquid‐crystalline phase with birefringence under crossed polarizers due to local orientation without aggregation.^[^ ^43^ ^]^ By performing our kneading method, MXene flakes that contain abundant surface functional groups (where T_x_ can be –F, –O, and –OH) can be tightly anchored to the VEH matrix through various interactions (e.g., hydrogen bonds), and will not leak even after 24 h of storage in water (Figure 3c, kneaded MXene content: 5 wt%). The resulting viscoelastic hydrogel/MXene composite (VEH‐MXene) displays very impressive mechanical properties. As Figure 3d and Movie S1 (Supporting Information) illustrate, an elliptical piece of VEH‐MXene with a length of 1.1 cm can be easily stretched to almost 121 cm, with a remarkably large elongation of >100 without fracture. To the best of our knowledge, such large stretchability has rarely been reported in conductive hydrogels.^[^ ^32^ ^]^ As the reference sample, the pristine viscosity‐dominated VEH cannot self‐support when stretched to a longer length (beyond 5000% strain) and often cause breakage due to relatively weak mechanical property (Movie S2, Supporting Information). In the rheological investigation, both G′ and G″ are enhanced by kneaded MXene (Figure 3e). The improvement of VEH‐MXene could be attributed to the homogeneous dispersion of MXene flakes as well as the formation of hierarchical cross‐linking structures within the polymer system. Although several works have reported the synthesis of PAA/ACC/MXene composite hydrogels by pre‐mixing MXene suspension with PAA/CaCl_2_ solution and then adding Na_2_CO_3_,^[^ ^40^, ^44^ ^]^ the negatively charged MXene inevitably flocculates in the presence of either monovalent (i.e., Na^+^) or divalent (i.e., Ca^2+^) cations due to the strong electrostatic interaction,^[^ ^45^ ^]^ thus may give rise to adverse agglomerates (Figure S6, Supporting Information). To mitigate this impact, prolonged mixing, such as stirring overnight, is often required.^[^ ^40^ ^]^ SEM images of VEH‐MXene just after mixing (Figure 3f) and after kneading and resting (Figure 3g) further reveal that our kneading method effectively diminishes the tendency of aggregating (indicated by blue circles) and facilitates the homogeneous dispersion of nanomaterials, which is also clearly evident by the uniform distribution of Ti elements from EDS images (Figure 3h). Particularly, XRD results show the same diffraction peaks of VEH‐MXene and MXene, proving that the kneading method can well maintain the crystal integrity of MXene (Figure 3i). The (002) diffraction peak of VEH‐MXene slightly downshifts from 6.16° to 5.94° with an enlarged interlayer spacing, which is attributed to various interactions between VEH and kneaded MXene, so that polymers serves as spacers to suppress the aggregation of MXenes as well as weaken the interplane attraction.^[^ ^46^ ^]^

With a resistive circuit design, the as‐prepared VEH‐MXene could report mechanical stimuli such as stretching to mimic mechano‐receptor of natural skins. Figure 3j shows the increased resistance as a function of axial strain applied, which is due to increased lateral distance and degradation of the percolation network of MXene flakes during the stretching process. The gauge factor (GF = (R−R_0_)/R_0_ *ε*, where *ε* is the strain) experiences a slow gradient increase in the strain range of 800%, with a maximum GF of 9.5.

In addition to MXene flakes, this kneading method is also applicable to GO slurry (Figure 3k and Figure S7a,b, Supporting Information). The resulting viscoelastic hydrogel/GO (VEH‐GO) shows remarkable malleability, uniform distribution, and reinforced modulus (Figure 3l and Figure S7c,d, Supporting Information).

**Figure 4:**
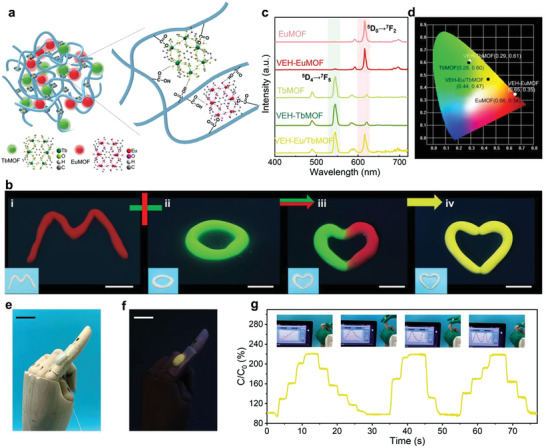
a) Schematic diagram of VEH‐LnMOF. b) Photographs showing the vivid emission colors of i) VEH‐EuMOF (red “M”), ii) VEH‐TbMOF (green “O”), iii) combined VEH‐EuMOF and VEH‐TbMOF (half red and half green “heart”), and iv) VEH‐ Eu/TbMOF (yellow “heart”) under UV light irradiation (*λ*_exc_ = 254 nm). The inset photos are taken under normal light. Scale bars: 1 cm. c) Fluorescence emission spectra and corresponding d) CIE 1931 chromaticity diagram of EuMOF, VEH‐EuMOF, TbMOF, VEH‐TbMOF, and VEH‐Eu/TbMOF, respectively. Photographs of the VEH‐Eu/TbMOF‐based skin‐like iontronics attached to a prosthetic finger with the assistance of VHB tape e) under daylight and f) under UV light irradiation (*λ*_exc_ = 254 nm). Scale bars: 2 cm. g) Real‐time capacitance changes when a finger is bent at different angles. The inset photos are from Movie S3 (Supporting Information).

As a demonstration of our kneading method for 3D materials, we take lanthanide metal‐organic frameworks (LnMOFs) composed of metal clusters and organic ligands as examples because of their sharp emission bands, vivid color purity, and substantial Stokes shifts.^[^ ^47^ ^]^ We first fabricate a kind of unique photoluminescent LnMOFs, Ln_2_(Mellitic)^.^6H_2_O according to previous reports,^[^ ^48^, ^49^ ^]^ where Ln is Eu^3+^ or Tb^3+^, producing EuMOFs with vivid red fluorescence or TbMOFs with green fluorescence. Their fluorescent emission spectra show the typical narrow characteristic peaks of EuMOFs (615 nm) and TbMOFs (545 nm), which corresponds to the Eu^3+ 5^D_0_ → ^7^F_2_ and Tb^3+ 5^D_4_ → ^7^F_5_ transitions, respectively (**Figure** **4**c).

Due to the multiple interactions, including chelation of lanthanide ions as well as hydrogen bonding between ligands and PAA (Figure 4a), the incorporation of LnMOFs into VEH by hand kneading results in fluorescent hydrogels with tunable photoluminescence properties while maintaining shapeable and autonomous self‐healing. As a result, the resultant viscoelastic hydrogel/EuMOF composite (VEH‐EuMOF) displays vivid red fluorescence and typical emission of Eu^3+^ ions, and has a very close Commission Internationale de L'Eclairage 1931 (CIE) coordinate (0.65, 0.35) to the pristine EuMOFs (0.66, 0.34) (Figure 4b‐i,c,d). Similarly, the viscoelastic hydrogel/TbMOF composite (VEH‐TbMOF) that can be manually shaped into a letter “O” also exhibits bright green fluorescence, typical emission of Tb^3+^ ions, and a very close CIE coordinate (0.29, 0.61) to the pristine TbMOF (0.28, 0.60) (Figure 4b‐ii,c,d). When two parts of VEH‐EuMOF and VEH‐TbMOF are gently attached, the interface can heal instantaneously, benefitting from various dynamic bonds (Figure 4b‐iii). Further mixing these two parts by kneading results in VEH‐Eu/TbMOF with uniform emission color of yellow under single‐wavelength excitation at 254 nm (Figure 4b‐iv), even when stretched (Figure S8, Supporting Information). The homogeneous dispersion of LnMOF crystals in the polymer matrix can be confirmed by SEM images (Figure S9, Supporting Information). The characteristic emissions of Ln^3+^ and Tb^3+^ ions are well retained (Figure 4c), and the corresponding CIE coordinates of VEH‐Eu/TbMOF (0.44,0.47) approaches to the yellow region (Figure 4d), which corroborates that the emission color of the composite is readily tuned by the types or ratios of kneaded LnMOFs. Moreover, their colors are the same under sunlight, showing promise for the potential application in anti‐counterfeiting fields.

In the presence of slightly dissolved free Ca^2+^ from ACC, VEH‐Eu/TbMOF is ionically conductive and thus enables the fabrication of a skin‐like capacitive sensor by integrating two hydrogel films with a dielectric layer (VHB tape), as shown in Figure 4e,f. When attached to a prosthetic hand, it fits well with the irregular shape of the prosthetic finger and can detect the bending movement of the prosthetic finger through real‐time feedback of capacitive signals (Figure 4g and Movie S3, Supporting Information).

As discussed above, 1D, 2D, and 3D nanomaterials have been demonstrated as “condiments” for designing and fabricating bionic skins with customizable functions. Nevertheless, the features and improvements offered by the individual filler are not sufficient for more complex requirements. As such, similar to the combination of “condiments” in kneading dough, the combination of various functional fillers may contribute to more tailored and integrated features. For example, to edit the properties of as‐prepared VEH‐MXene, TbMOFs (5 wt%) with green fluorescence were further introduced by kneading to make it have both electrical and fluorescent properties. The resulting VEH‐MXene‐TbMOF has a similar appearance to VEH‐MXene under daylight (Figure S10a, Supporting Information). More visually, when integrated into a closed battery‐powered circuit, it can act as a part of the wire to light a blue LED, while displaying vivid green fluorescence under UV light irradiation (*λ*_exc_ = 254 nm, Figure S10b,c, Supporting Information). Moreover, our kneading method has excellent flexibility in the addition order of fillers.

**Figure 5:**
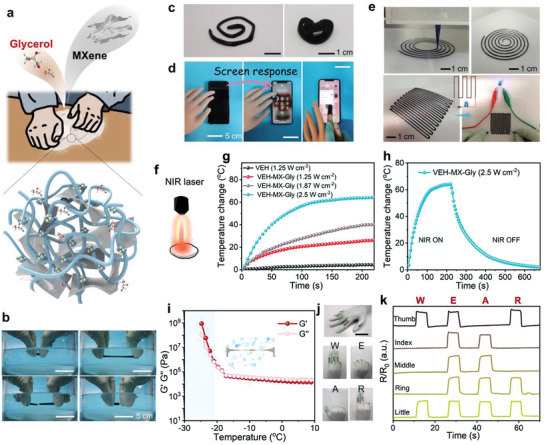
a) Schematic diagram of the preparation of VEH‐MX‐Gly. b) Underwater self‐healing capacity of VEH‐MX‐Gly. c) Photographs demonstrating the malleability of VEH‐MX‐Gly. d) The prosthetic finger coated by VEH‐MX‐Gly is responsive when touched to the screen. e) 3D printed VEH‐MX‐Gly as the conductive wire for lighting a LED. f) The NIR photothermal property of VEH‐MX‐Gly. g) Curves of temperature change versus time under 808 nm laser irradiation with different laser power densities. h) Photothermal effect of the VEH‐MX‐Gly with a power density of 2.5 W cm^−2^ at 808 nm and the laser is then switched off after irradiation for 210 s. i) G′ and G″ of VEH‐MX‐Gly as a function of temperature. j) Photographs of a “data glove” mounted with the assembled skin‐like sensors to perform a series of gestures. Scale bar: 5 cm. k) Relative resistance changes of skin‐like sensors in response to sign languages.

From the perspective of practical applications of biomimetic skin‐like materials, a series of highly integrated properties including high stretchability, mechanical adaptability, self‐healing ability, electrical conductivity, sensory capabilities, freezing tolerance and ease processability are prerequisites, which can be readily achieved by kneading MXene (5 wt%) and glycerol (15 wt%) into the VEH matrix, as depicted by **Figure** **5**a. Benefitting from the synergy between polymer matrix and fillers, the obtained viscoelastic hydrogel/MXene/glycerol composites (VEH‐MX‐Gly) exhibits high stretchability up to 8000% (Figure S11, Supporting Information), great malleability and self‐healing property both in ambient and aquatic conditions (Figure 5b, Figure S12 and Movie S4, Supporting Information). It can be remolded into diverse desired shapes, such as a coil and a heart shape (Figure 5c). The mechanical adaptability enables VEH‐MX‐Gly to wrap around the irregular surface of a prosthetic finger, which helps the prosthetic finger to operate a smartphone smoothly. In contrast, a prosthetic hand made of rubber is incapable of controlling smartphones (Figure 5d). As shown in Figure S13 (Supporting Information), the VEH‐MX‐Gly shows typical shear‐thinning behavior, making it advantageous to be applied in 3D printing (direct ink writing). The lower viscosity under higher shear rates renders the easy flow of VEH‐MX‐Gly through the fine nozzle during printing, while the high viscosity under low shear rates allows high fidelity and shape retention after extrusion. Accordingly, the VEH‐MX‐Gly can be facilely processed and directly printed on different substrates to construct customizable structures (Figure 5e and Movie S5, Supporting Information). We demonstrated a soft circuit whereby the LED lights up in the ambient environment, and the presence of glycerol enhances its water retention.

In addition, the introduction of MXene also endows the composite hydrogel with outstanding near‐infrared (NIR) photothermal performance (Figure 5f).^[^ ^50^, ^51^ ^]^ Temperature‐time profiles for different optical powers are shown in Figure 5g,h, the VEH‐MX‐Gly exhibits an obvious photothermal‐conversion behavior, its temperature elevation is 25–63 °C within 250 s under 808 nm laser irradiation at power density ranging from 1.25 to 2.5 W cm^–2^. In contrast, pristine VEH shows no significant temperature change under the same condition, indicating that the photothermal performance of MXene nanosheets is well retained by kneading in the composite hydrogels. Besides, the incorporation of glycerol into the hydrogel network has been proved to inhibit the water crystallization at sub‐zero temperature by lowering the freezing point.^[^ ^52^ ^]^ The pristine VEH shows an abrupt increase in G′ and G″ when the temperature decreases near −10 °C (Figure S14, Supporting Information), while G′ and G″ of VEH‐MX‐Gly maintain an almost constant value as the temperature drops below −20 °C, revealing the satisfactory anti‐freezing performance (Figure 5i). Compared with most malleable hydrogels, the VEH‐MX‐Gly material offers significant advantages in terms of stretchability, freezing tolerance, self‐healability and 3D printability (Table S1, Supporting Information).

With the combined advantages, a data glove prototype was further fabricated by implanting printed VEH‐MX‐Gly‐based sensors on a glove for communicating the message of sign language. When the data glove is worn, the bending of the finger drives the sensor to bend, resulting in increased resistance of the VEH‐MX‐Gly‐based sensor. In this way, the word “WEAR” can be successfully transmitted (Figure 5j,k).

Moreover, to demonstrate the good flexibility of our kneading method in the order of filler addition, VEH‐Gly‐MX was prepared in a different addition order by kneading glycerol followed by MXene. Interestingly, both VEH‐Gly‐MX and VEH‐MX‐Gly display the almost identical appearance and electrical conductivity (Figure S15, Supporting Information). In addition, the rheological results show that the two samples have comparable moduli (G′ and G″) and can maintain an almost constant value as the temperature drops below −20 °C, indicating the good flexibility of our kneading method.

## 3 Conclusion

In conclusion, we have reported a universal and straightforward strategy, the so‐called kneading method containing two steps of kneading and resting. This kneading method has three critical features allowing universal design and fabrication of biomimetic skins with customized functions: (1) facile, (2) homogeneous, and (3) making full use of raw materials without any waste. To demonstrate the versatility of this kneading method, we thus investigated the dispersion homogeneity and new exciting functions of four skin‐like conductive hydrogels by kneading typical 1D, 2D, 3D nanomaterials into a kind of viscoelastic hydrogel alone or in combination form. Since kneading promotes homogeneous mixing of all the ingredients, and the subsequent resting is advantageous to autonomous regulation and optimization of the network structure. Indeed, this “add‐on” approach is conductive to yielding desired functionalities derived from one or more “condiments,” which includes but not limited to mechanical compliance, sensory capabilities, freezing resistance, 3D printability, photothermal responsiveness, and fluorescence tunability, while preserving the viscoelasticity of the host hydrogel. The facileness and reliability of the proposed method make the biomimetic skin‐like materials suitable for future mass fabrication and opens up a new path for broadening the scope of applications to more complex environments.

## Conflict of Interest

The authors declare no conflict of interest.

## Supporting information

Supporting Information

Supplemental Movie 1

Supplemental Movie 2

Supplemental Movie 3

Supplemental Movie 4

Supplemental Movie 5

## Data Availability

The data that support the findings of this study are available from the corresponding author upon reasonable request.

## References

[1] S. Wang , J. Y. Oh , J. Xu , H. Tran , Z. Bao , Acc. Chem. Res. 2018, 51, 1033. 29693379 10.1021/acs.accounts.8b00015

[2] A. Chortos , J. Liu , Z. Bao , Nat. Mater. 2016, 15, 937. 27376685 10.1038/nmat4671

[3] M. Wang , Y. Luo , T. Wang , C. Wan , L. Pan , S. Pan , K. He , A. Neo , X. Chen , Adv. Mater. 2021, 33, 2003014. 10.1002/adma.202003014 32930454

[4] B. Xue , H. Sheng , Y. Li , L. Li , W. Di , Z. Xu , L. Ma , X. Wang , H. Jiang , M. Qin , Z. Yan , Q. Jiang , J.‐M. Liu , W. Wang , Y. Cao , Natl. Sci. Rev. 2021, 10.1093/nsr/nwab147. PMC9375542 35974839

[5] S. Chen , L. Sun , X. Zhou , Y. Guo , J. Song , S. Qian , Z. Liu , Q. Guan , E. M. Jeffries , W. Liu , Y. Wang , C. He , Z. You , Nat. Commun. 2020, 11, 1107. 32107380 10.1038/s41467-020-14446-2 PMC7046662

[6] C. M. Tringides , N. Vachicouras , I. de Lázaro , H. Wang , A. Trouillet , B. R. Seo , A. Elosegui‐Artola , F. Fallegger , Y. Shin , C. Casiraghi , K. Kostarelos , S. P. Lacour , D. J. Mooney , Nat. Nanotechnol. 2021, 16, 1019. 34140673 10.1038/s41565-021-00926-z PMC9233755

[7] H.‐R. Lee , C.‐C. Kim , J.‐Y. Sun , Adv. Mater. 2018, 30, 1704403. 10.1002/adma.201704403 29889329

[8] Z. Lei , J. Huang , P. Wu , Adv. Funct. Mater. 2020, 30, 1908018.

[9] T. Li , Y. Wang , S. Li , X. Liu , J. Sun , Adv. Mater. 2020, 32, 2002706. 10.1002/adma.202002706 32589326

[10] Z. Liu , Y. Wang , Y. Ren , G. Jin , C. Zhang , W. Chen , F. Yan , Mater. Horiz. 2020, 7, 919.

[11] M. Baumgartner , F. Hartmann , M. Drack , D. Preninger , D. Wirthl , R. Gerstmayr , L. Lehner , G. Mao , R. Pruckner , S. Demchyshyn , L. Reiter , M. Strobel , T. Stockinger , D. Schiller , S. Kimeswenger , F. Greibich , G. Buchberger , E. Bradt , S. Hild , S. Bauer , M. Kaltenbrunner , Nat. Mater. 2020, 19, 1102. 32541932 10.1038/s41563-020-0699-3

[12] V. R. Feig , H. Tran , M. Lee , Z. Bao , Nat. Commun. 2018, 9, 2740. 30013027 10.1038/s41467-018-05222-4 PMC6048132

[13] Y. Zhou , C. Wan , Y. Yang , H. Yang , S. Wang , Z. Dai , K. Ji , H. Jiang , X. Chen , Y. Long , Adv. Funct. Mater. 2019, 29, 1806220.

[14] H. Li , G. Gao , Z. Xu , D. Tang , T. Chen , Macromol. Rapid Commun. 2021, 42, 2100480. 10.1002/marc.202100480 34505726

[15] Z. Lei , P. Wu , Acc. Mater. Res. 2021, 2, 1203.

[16] T. Long , Y. Li , X. Fang , J. Sun , Adv. Funct. Mater. 2018, 28, 1804416.

[17] Y. Shang , C. Wu , C. Hang , H. Lu , Q. Wang , Adv. Mater. 2020, 32, 2000189. 10.1002/adma.202000189 32567056

[18] H. Liu , H. Zhang , W. Han , H. Lin , R. Li , J. Zhu , W. Huang , Adv. Mater. 2021, 33, 2004782. 10.1002/adma.202004782 33448066

[19] Y. Xu , R. Rothe , D. Voigt , S. Hauser , M. Cui , T. Miyagawa , M. Patino Gaillez , T. Kurth , M. Bornhäuser , J. Pietzsch , Y. Zhang , Nat. Commun. 2021, 12, 2407. 33893308 10.1038/s41467-021-22675-2 PMC8065207

[20] G. Ye , J. Qiu , X. Fang , T. Yu , Y. Xie , Y. Zhao , D. Yan , C. He , N. Liu , Mater. Horiz. 2021, 8, 1047. 34821335 10.1039/d0mh01656j

[21] Y. Wang , C. Zhu , R. Pfattner , H. Yan , L. Jin , S. Chen , F. Molina‐Lopez , F. Lissel , J. Liu , N. I. Rabiah , Z. Chen , J. W. Chung , C. Linder , M. F. Toney , B. Murmann , Z. Bao , Sci. Adv. 2017, 3, e1602076. 28345040 10.1126/sciadv.1602076 PMC5345924

[22] G. Gao , F. Yang , F. Zhou , J. He , W. Lu , P. Xiao , H. Yan , C. Pan , T. Chen , Z. L. Wang , Adv. Mater. 2020, 32, 2004290. 10.1002/adma.202004290 33174265

[23] Y.‐Z. Zhang , J. K. El‐Demellawi , Q. Jiang , G. Ge , H. Liang , K. Lee , X. Dong , H. N. Alshareef , Chem. Soc. Rev. 2020, 49, 7229. 32936169 10.1039/d0cs00022a

[24] H. Peng , Y. Xin , J. Xu , H. Liu , J. Zhang , Mater. Horiz. 2019, 6, 618.

[25] M. Ma , Y. Shang , H. Shen , W. Li , Q. Wang , Chem. Eng. J. 2021, 420, 129865.

[26] Y. Xu , P. A. Patsis , S. Hauser , D. Voigt , R. Rothe , M. Günther , M. Cui , X. Yang , R. Wieduwild , K. Eckert , C. Neinhuis , T. F. Akbar , I. R. Minev , J. Pietzsch , Y. Zhang , Adv. Sci. 2019, 6, 1802077. 10.1002/advs.201802077 PMC6685503 31406658

[27] M. Wang , Z. Lai , X. Jin , T. Sun , H. Liu , H. Qi , Adv. Funct. Mater. 2021, 31, 2101957.

[28] J. Song , S. Chen , L. Sun , Y. Guo , L. Zhang , S. Wang , H. Xuan , Q. Guan , Z. You , Adv. Mater. 2020, 32, 1906994. 10.1002/adma.201906994 31957099

[29] O. Parenti , L. Guerrini , B. Zanoni , M. Marchini , M. G. Tuccio , E. Carini , Food Chem. 2021, 338, 128120. 33091998 10.1016/j.foodchem.2020.128120

[30] O. Parenti , L. Guerrini , S. B. Mompin , M. Toldrà , B. Zanoni , J. Food Eng. 2021, 309, 110692.

[31] C. S. Boland , U. Khan , G. Ryan , S. Barwich , R. Charifou , A. Harvey , C. Backes , Z. Li , M. S. Ferreira , M. E. Möbius , R. J. Young , J. N. Coleman , Science 2016, 354, 1257. 27940866 10.1126/science.aag2879

[32] Y.‐Z. Zhang , K. H. Lee , D. H. Anjum , R. Sougrat , Q. Jiang , H. Kim , H. N. Alshareef , Sci. Adv. 2018, 4, 0098. 10.1126/sciadv.aat0098 PMC6003726 29922718

[33] S. Sun , L.‐B. Mao , Z. Lei , S.‐H. Yu , H. Cölfen , Angew. Chem., Int. Ed. 2016, 55, 11765. 10.1002/anie.201602849 27444970

[34] M. Lin , Z. Zheng , L. Yang , M. Luo , L. Fu , B. Lin , C. Xu , Adv. Mater. 2021, 34, 2107309. 10.1002/adma.202107309 34648668

[35] D. J. Schupp , X. Zhang , S. Sun , H. Cölfen , Chem. Commun. 2019, 55, 4913. 10.1039/c8cc08986h 30887968

[36] Z. Lei , P. Wu , Nat. Commun. 2019, 10, 3429. 31366932 10.1038/s41467-019-11364-w PMC6668389

[37] Z. Lei , S. Sun , P. Wu , ACS Sustainable Chem. Eng. 2017, 5, 4499.

[38] S. C. Lee , G. Gillispie , P. Prim , S. J. Lee , Chem. Rev. 2020, 120, 10834. 32815369 10.1021/acs.chemrev.0c00015 PMC7673205

[39] A. VahidMohammadi , J. Rosen , Y. Gogotsi , Science 2021, 372, 1581. 10.1126/science.abf1581 34112665

[40] X. Li , L. He , Y. Li , M. Chao , M. Li , P. Wan , L. Zhang , ACS Nano 2021, 15, 7765. 33769046 10.1021/acsnano.1c01751

[41] Y. Zhang , M. Gong , P. Wan , Matter 2021, 4, 2655.

[42] X. Huang , P. Wu , Adv. Funct. Mater. 2020, 30, 1910048.

[43] W. Eom , H. Shin , R. B. Ambade , S. H. Lee , K. H. Lee , D. J. Kang , T. H. Han , Nat. Commun. 2020, 11, 2825. 32499504 10.1038/s41467-020-16671-1 PMC7272396

[44] Y. Zhu , J. Liu , T. Guo , J. J. Wang , X. Tang , V. Nicolosi , ACS Nano 2021, 15, 1465. 33397098 10.1021/acsnano.0c08830

[45] Y. Deng , T. Shang , Z. Wu , Y. Tao , C. Luo , J. Liang , D. Han , R. Lyu , C. Qi , W. Lv , F. Kang , Q.‐H. Yang , Adv. Mater. 2019, 31, 1902432. 10.1002/adma.201902432 31513318

[46] Z. Wu , T. Shang , Y. Deng , Y. Tao , Q.‐H. Yang , Adv. Sci. 2020, 7, 1903077. 10.1002/advs.201903077 PMC7141041 32274307

[47] S. Abednatanzi , P. Gohari Derakhshandeh , H. Depauw , F.‐X. Coudert , H. Vrielinck , P. Van Der Voort , K. Leus , Chem. Soc. Rev. 2019, 48, 2535. 30989162 10.1039/c8cs00337h

[48] J. Huang , P. Wu , Nano‐Micro Lett. 2020, 13, 15. 10.1007/s40820-020-00543-w PMC8187549 34138212

[49] L. L. da Luz , R. Milani , J. F. Felix , I. R. B. Ribeiro , M. Talhavini , B. A. D. Neto , J. Chojnacki , M. O. Rodrigues , S. A. Júnior , ACS Appl. Mater. Interfaces 2015, 7, 27115. 26523753 10.1021/acsami.5b06301

[50] W. Tang , Z. Dong , R. Zhang , X. Yi , K. Yang , M. Jin , C. Yuan , Z. Xiao , Z. Liu , L. Cheng , ACS Nano 2019, 13, 284. 30543399 10.1021/acsnano.8b05982

[51] K. Li , T.‐H. Chang , Z. Li , H. Yang , F. Fu , T. Li , J. S. Ho , P.‐Y. Chen , Adv. Energy Mater. 2019, 9, 1901687.

[52] F. Chen , D. Zhou , J. Wang , T. Li , X. Zhou , T. Gan , S. Handschuh‐Wang , X. Zhou , Angew. Chem., Int. Ed. 2018, 57, 6568. 10.1002/anie.201803366 29656553

