# Supplementary material for: Kneading‐Inspired Versatile Design for Biomimetic Skins with a Wide Scope of Customizable Features
Source: Adv Sci (Weinh). 2022 Mar 22;9(14):2200108. doi: 10.1002/advs.202200108 (PMC9109062; doi:10.1002/advs.202200108)
Supplement: Supplementary file 1 — Supporting Information [file ADVS-9-2200108-s002.pdf]

Supporting Information

Kneading-Inspired Versatile Design for Biomimetic Skins with a Wide Scope of Customizable Features

*Jiahui Huang, and Peiyi Wu**

E-mail: peiyiwu@fudan.edu.cn

Experimental Section

Materials

Polyacrylic acid (PAA, Mw ≈ 100,000 g mol^{-1}) was obtained from Sigma-Aldrich Co. Calcium chloride ($CaCl_2$), europium(III) nitrate hexahydrate ($Eu(NO_3)_3 \cdot 6H_2O$), terbium(III) nitrate pentahydrate ($Tb(NO_3)_3 \cdot 5H_2O$), and mellitic acid were purchased from Aladdin Chemical Co. Sodium carbonate (Na_2CO_3), ethanol, and glycerol were purchased from Sinopharm Chemical Reagent Co. Carbon nanotubes slurry (CNTs) with a concentration of 4.3 wt% were purchased from Qingdao Haoxin Co. Ltd (China). Ti_3AlC_2 (MAX) powder with the average particle size of 200 meshes was purchased from Jilin 11 Technology Co., Ltd. Hydrofluoric (HF), hydrochloric (HCl), and sulfuric acid (H_2SO_4) were got from Shanghai Chemical Corp. Graphene oxide (GO) nanosheets were purchased from Times Nano (China). All reagents were used without further purification.

Preparation of the viscoelastic hydrogel (VEH)

According to the previous report,[S1] 0.1 M Na_2CO_3 solution was slowly (*ca*. 5 mL min^{-1}) added to a mixed solution of PAA (0.1 M) and $CaCl_2$ (0.1 M) with vigorous stirring. Then, a white sticky hydrogel gradually formed around the stirring bar. After stirring for another hour, the turbid solution was discarded. PAA-ACC viscoelastic hydrogel was finally obtained after being washed with deionized water several times.

Preparation of delaminated MXene slurry

Delaminated MXene slurry was prepared according to our previously reported freeze-and-thaw (FAT) method.[S2] After four cycles of FAT, the precipitate consisting of unreacted MAX and multilayer MXene was discarded after centrifugation at 1500 rpm for 30

min, and the suspension containing the delaminated MXene flakes was collected after centrifugation at 3500 rpm for 30 min, which termed as delaminated MXene solution. MXene slurry was finally obtained by centrifuging delaminated MXene solution at 9000 rpm for 60min, whose concentration was calculated at about 40 mg/mL.

Preparation of Lanthanide Metal‑ Organic Frameworks (LnMOFs)

LnMOFs crystals with red fluorescence (EuMOFs) and green fluorescence (TbMOFs) were synthesized according to the previously reported rapid solvent precipitation method.[S3, S4] Specifically, to prepare EuMOFs crystals, 1 mmol $Eu(NO_3)_3 \cdot 6H_2O$, 1 mmol mellitic acid, and 10 mL deionized water were first magnetically stirred for 10 min. Then rapid crystal nucleation was triggered by the slow addition of 3 mL ethanol solvent (30% in volume). After several minutes, the formed crystals were collected by centrifugation at 5000 r.p.m. for 5 min. TbMOFs crystals were synthesized in a similar way, except that $Eu(NO_3)_3 \cdot 6H_2O$ was replaced by $Tb(NO_3)_3 \cdot 5H_2O$.

Kneading method

The kneading process is similar to kneading dough. Taking VEH-CNT as an example, CNT paste was first spread on the as-prepared viscoelastic hydrogel (VEH), then our hands acted as an agitator to stir the ingredients. The mixed hydrogel was repeatedly rolled and flattened until its color became uniformly black. Note that the CNT paste should be added in small amounts with many times to achieve better homogeneity. To prepare large-sized VEH-CNT samples, an automatic household dough maker (HMJ-A20E1 2L) was used. For VEH-MXene, the MXene content was 5 wt%. For VEH-GO, the GO content was 5 wt%. For VEH-EuMOF, the content of kneaded EuMOF was 5 wt%. For VEH-TbMOF, the kneaded TbMOF was 5

wt%. For VEH-MX-Gly, the kneaded MXene was 5 wt% and the glycerol content was 15 wt%. VEH-MX-Gly was prepared by kneading MXene first and then glycerol, while VEH-Gly-MX was obtained by kneading glycerol followed by MXene.

Characterization

Fourier transforms infrared (FTIR) spectra were collected by Nicolet Nexus 470 spectrometer. Rheological properties of hydrogel samples were characterized at 25 ^{o}C using a HAAKE MARS modular advanced rheometer with a 25-mm parallel plate. The storage modulus (G′) and loss modulus (G″) were measured in the frequency range from 0.1 to 100 Hz at 0.1% strain amplitude. The temperature sweep measurement of the VEH-MX-Gly was measured with a temperature range of 10 to -25 °C, a fixed oscillatory strain of 0.1%, and a fixed frequency of 1 Hz. Scanning electron microscopy (SEM) images were recorded by field emission scanning electron microscopy (FESEM, Zeiss, Ultra 55), and the element mapping images were taken from the energy dispersive spectrometer (EDS) equipped on a FESEM (S-4800, Hitachi, Japan). The morphology and structure of MXene, GO, and CNT were observed by TEM (JEOL JEM2011 F Microscope). Fluorescent emission spectra were obtained with FLS 1000 at ambient conditions. X-ray diffraction (XRD) data were measured on Bruker D8 diffractometer (Germany) with Ni-filtered Cu Kα radiation (40 kV, 40 mA). Due to the excellent shape adaptation of VEH-CNT, the volume of VEH and VEH-CNT was measured with the syringe. The Teflon mold used for resistance test was shown in Figure S3, with two flexible conductive tapes adhered onto two sides. The hollow part of the mold was used to hold samples with dimensions of 1 × 1 × 1 cm^3. Capacitance and resistance signals were measured on an LCR meter (TH2830) with an AC voltage of 1V and a sweeping

frequency of 1 kHz. The conductivity (σ) was calculated from the formula, σ = L/RS, where L and S corresponded to the length and cross-sectional area of hydrogels, respectively. The VEH-MX-Gly was printed by a 3D printing system (3D Bio-Architect work station, Regenovo), with a tip diameter of 0.4 mm, extrusion pressure of 0.3 MPa, and printing speed of 4 mm s^{-1}. The entire printing environment was kept at about 25 ^{o}C. Photothermal effect measurements were performed under irradiation by 808 nm diode laser with a spot size of 5×8 mm^2 (Changchun New Industries Optoelectronics Technology Co., Ltd., MDL-III-808R), which was oriented perpendicular to hydrogel samples. And the variation of the temperature was recorded every 5 seconds by a thermocouple thermometer.

Supplementary Figures

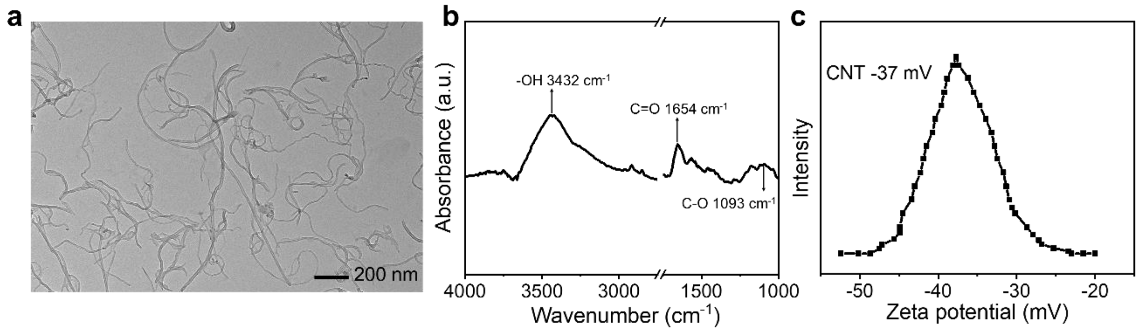

Figure S1. (a) TEM image of CNTs with a diameter of ~20 nm. (b) FTIR spectrum of CNTs. The characteristic peaks at 1093 cm^{-1} (C-O stretching modes), 1654 cm^{-1} (C=O stretching mode), 3432 cm^{-1} (-OH stretching band) suggest the existence of oxygen-containing functional groups at the CNT surfaces. (c) Zeta potential measurements of CNTs.

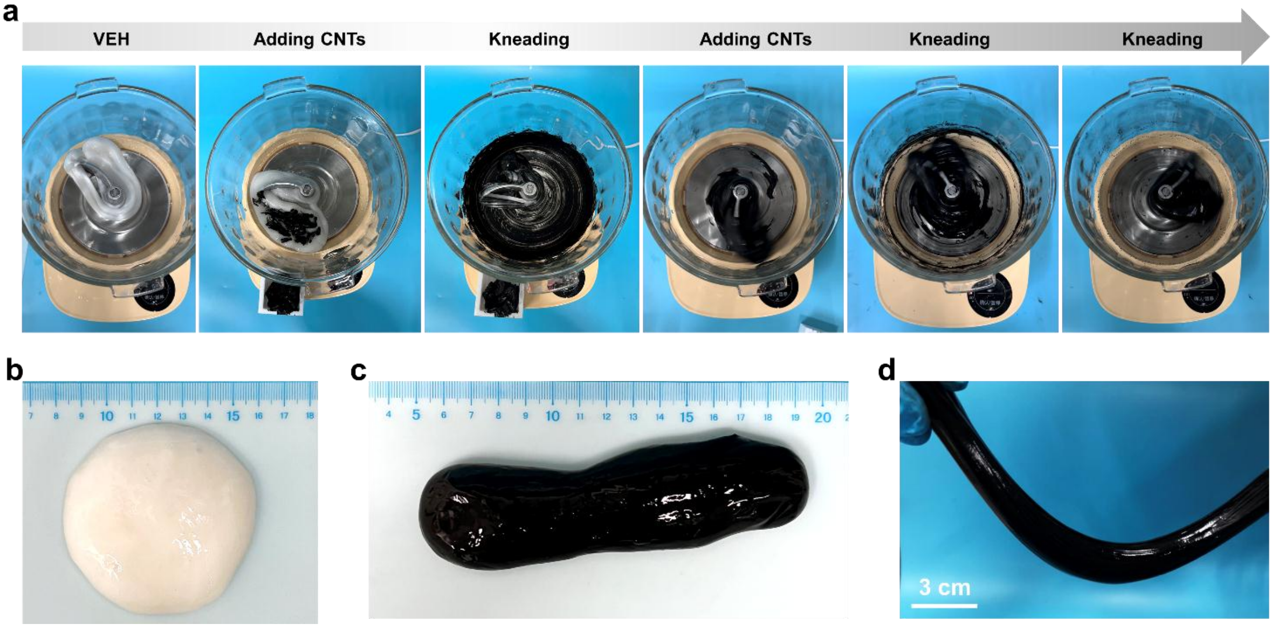

Figure S2. (a) Photographs of kneading process by an automatic household dough maker for preparing VEH-CNT with larger size. The weight of VEH is 90 g, and the CNT content is 1 wt%. (b) Photograph of pristine VEH. (c-d) Photographs of VEH-CNT with CNT content of 1 wt% after kneading and resting.

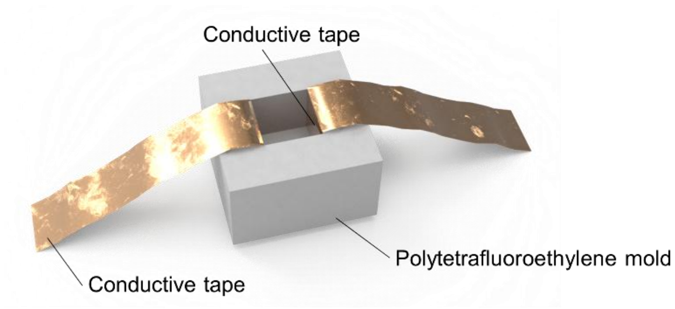

Figure S3. Schematic diagram of the Teflon (polytetrafluoroethylene) mold used to measure resistance.

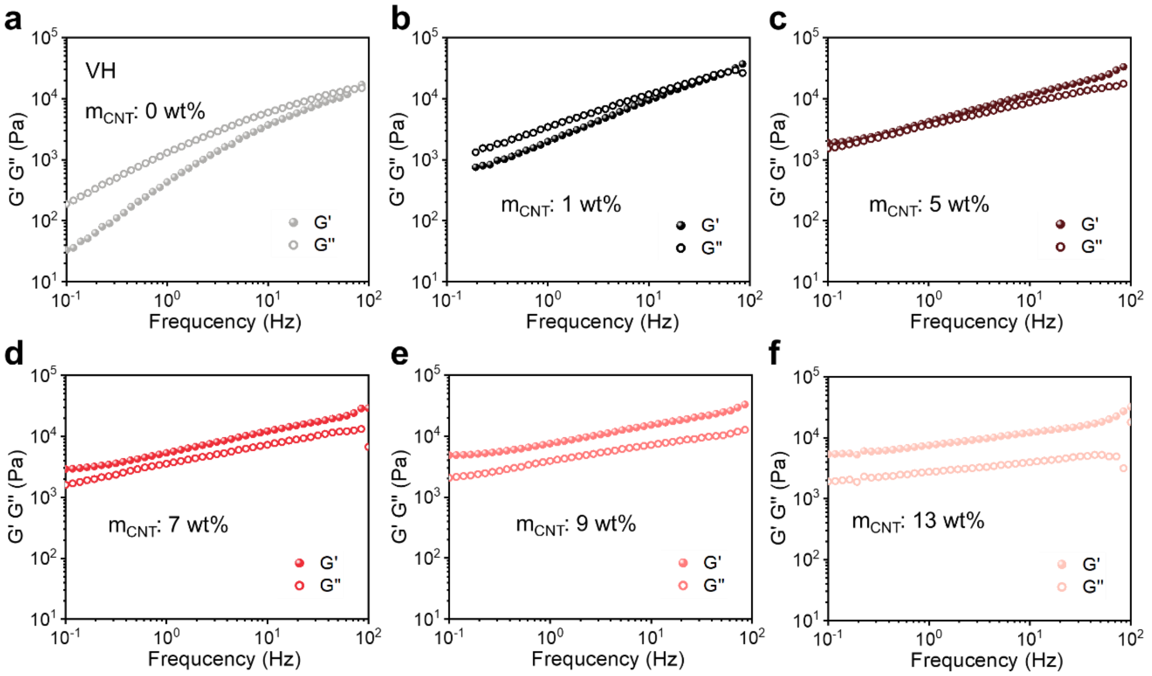

Figure S4. CNT content effect on the rheological behavior of VH-CNT. The CNT contents are 0, 1, 5, 7, 9, and 13 wt% in (a), (b), (c), (d), (e), and (f), respectively.

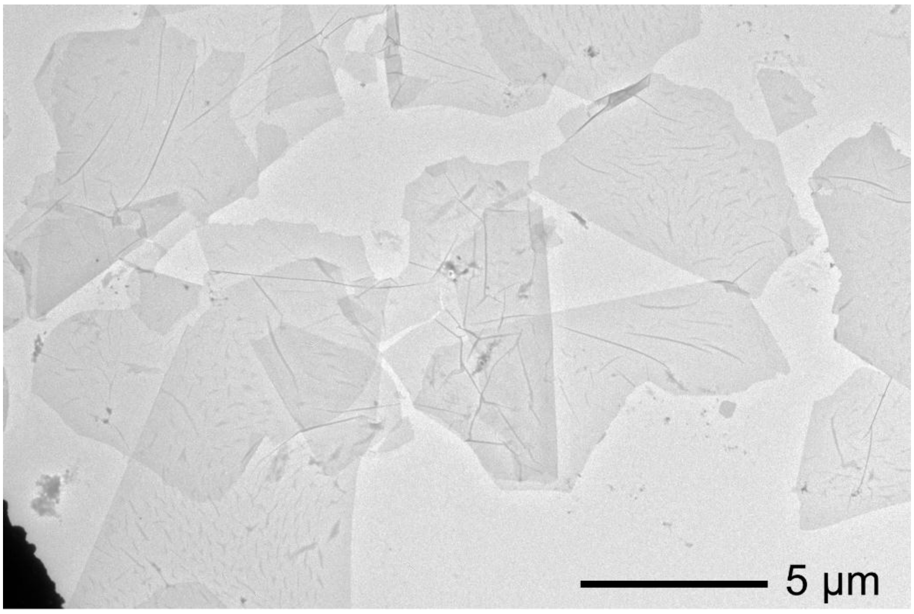

Figure S5. TEM image of large and high-quality MXene ($Ti_3C_2T_x$) flakes.

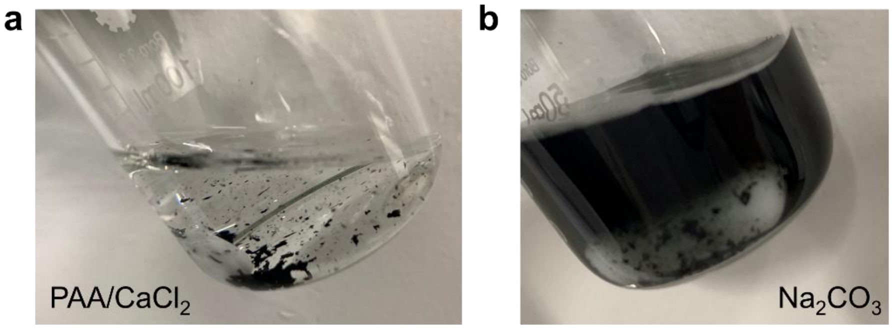

Figure S6. Photos showing the mixture of a small amount of MXene suspension with (a) 1 M PAA/$CaCl_2$ solution and (b) 1 M Na_2CO_3 solution.

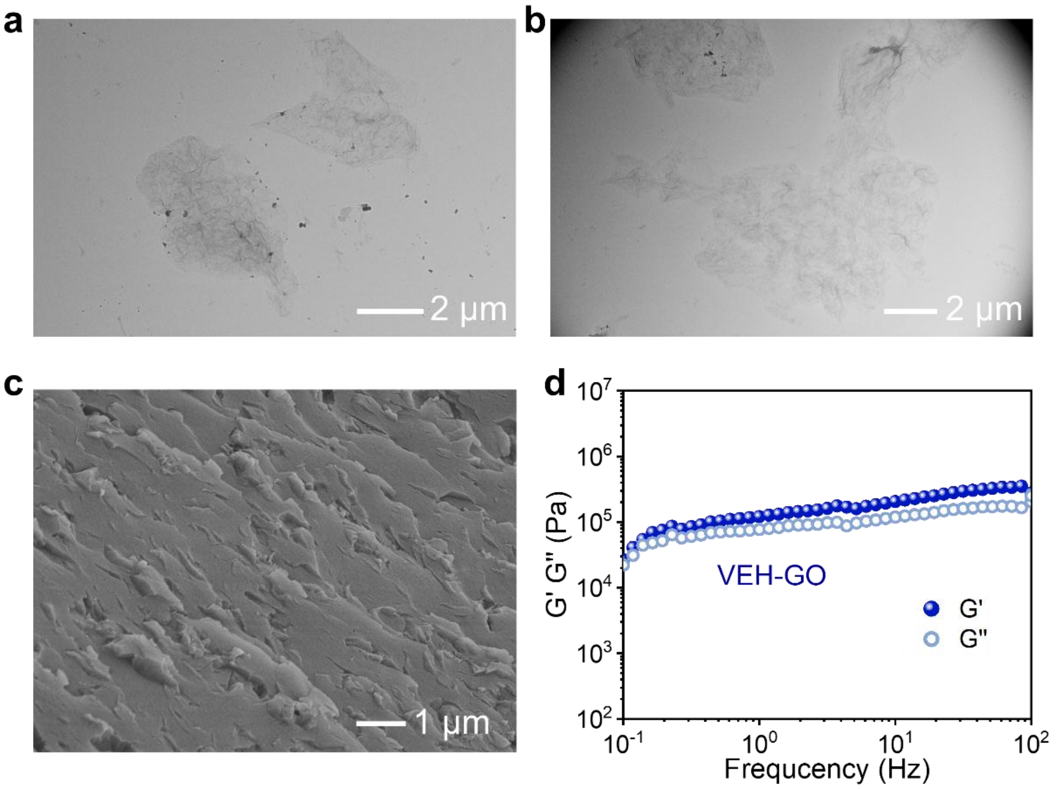

Figure S7. (a)-(b) TEM images of GO nanosheets with lateral size of ~1 μm. (c) SEM image of VEH-GO. (d) G′ and G″ moduli of VEH-GO.

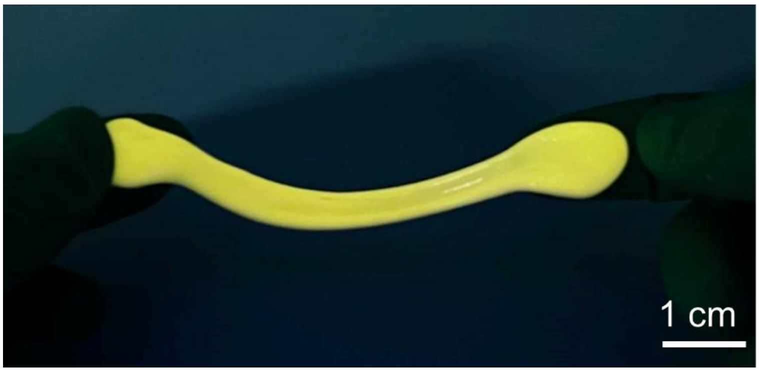

Figure S8. Photograph of VEH-Eu/TbMOF under UV light irradiation (λ_{exc} = 254 nm).

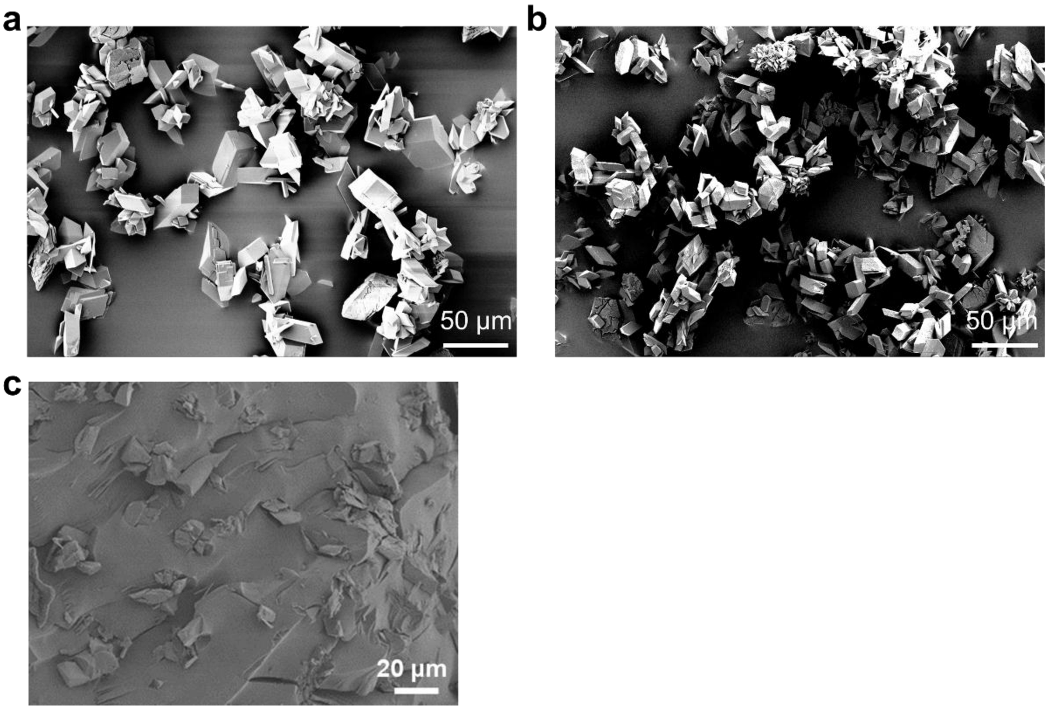

Figure S9. SEM images of (a) EuMOFs with ethanol as precipitant agents (b) TbMOFs as ethanol as precipitant and (c) VEH-Eu/TbMOF.

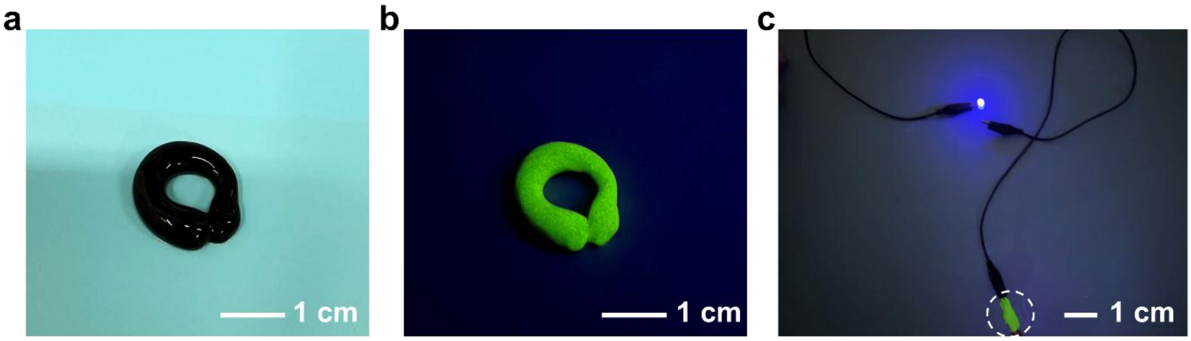

Figure S10. Photographs of VEH-MXene-TbMOF (a) under daylight and (b) under UV light irradiation (λ_{exc} = 254 nm). (c) Lighting a blue LED in a closed battery-powered circuit connected with VEH-MXene-TbMOF. Photograph was taken under UV irradiation (λ_{exc} = 254 nm).

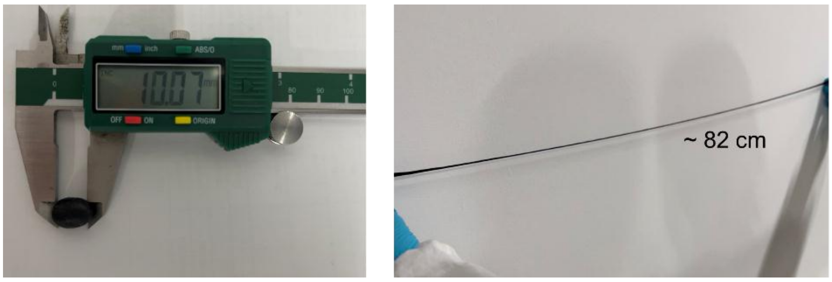

Figure S11. Stretchability of VEH-MX-Gly.

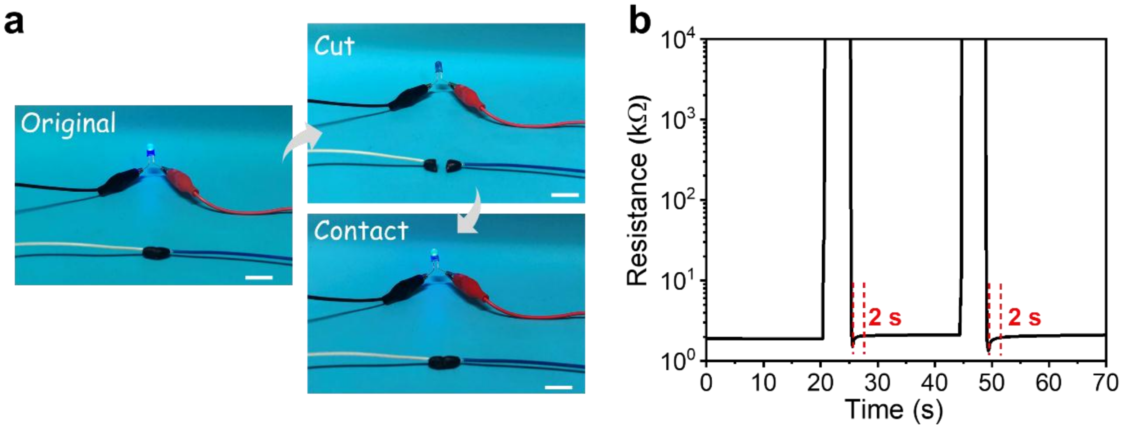

Figure S12. (a) Photographs of the self-healing process of VEH-MX-Gly in the ambient environment. (b) Electric self-healing process of VEH-MX-Gly recorded by real-time resistance in the ambient environment.

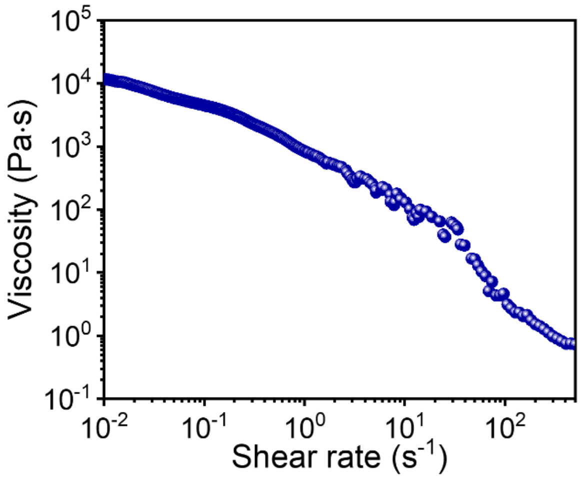

Figure S13. Shear-thinning behavior of VEH-MX-Gly.

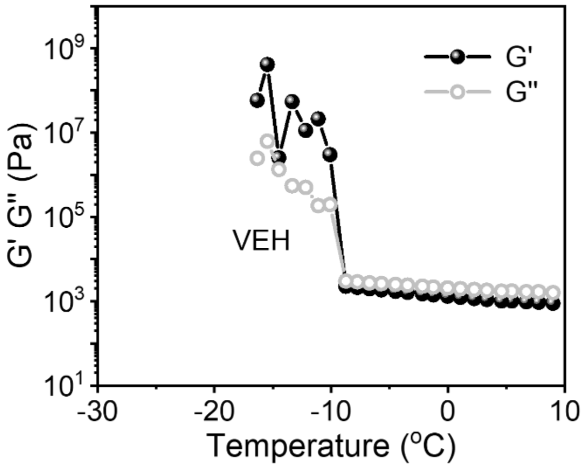

Figure S14. G′ and G″ of VEH as a function of temperature.

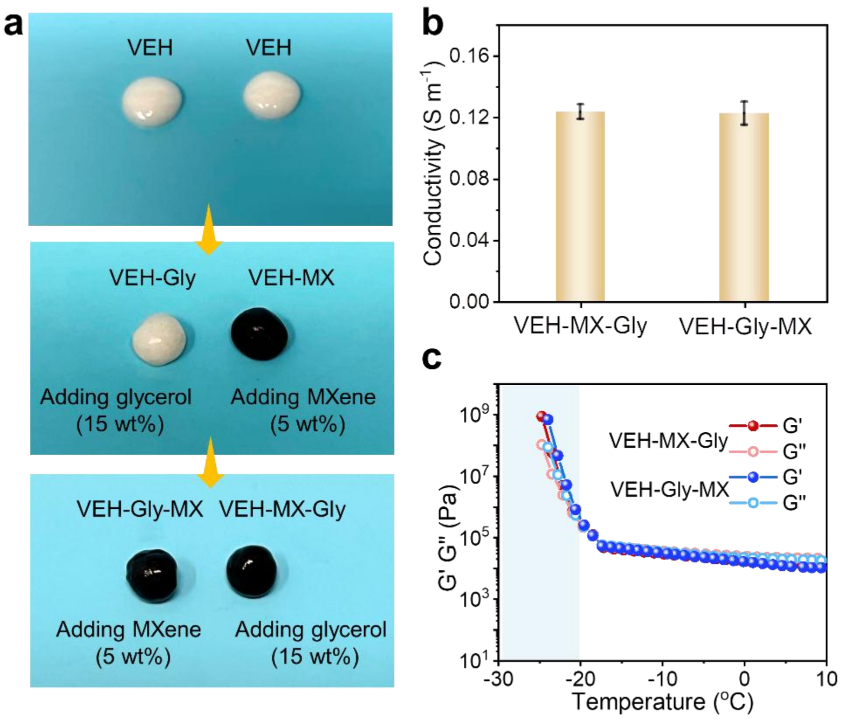

Figure S15. (a) Photographs of preparing VEH-Gly-MX and VEH-MX-Gly with different addition order. (b) Conductivity of VEH-MX-Gly and VEH-Gly-MX. (c) G′ and G″ of VEH-MX-Gly and VEH-Gly-MX as a function of temperature.

Table S1. Comparation of performance of malleable hydrogels.

Materials	Stretchability	Freezing tolerance	Self-healing efficiency	3D printability	Ref
PAA/PVA/Fe^{3+}/ borax/CNT/EG	550%	-25 °C	90.4%	/	[S5]
Dual nanocomposite hydrogel (DNC)	800%	/	/	/	[S6]
low-molecular-weight hyperbranched polyglycidol/PBA/PAAm	41600%	/	97.2	/	[S7]
PHEMA/PVA/borax	3330%	/	74%	/	[S8]
CareGum (PVA/TA/Fe^{3+}/PDA/HA)	30000%	/	100%	Yes	[S9]
Dopamine-triggered PSBMA hydrogel	350%	/	Almost 100%	Yes	[S10]
PPVA/pyrrole	~800%	/	96%	Yes	[S11]
PNIPAM/Laponite/CNT	1200%	/	Almost 100%	Yes	[S12]
Ca-PAA-SA-CNTs.	~600%	/	Almost 100%	Yes	[S13]
PAA/ACC/MXene	1200%	/	Almost 100%	/	[S14]
VEH-MX-Gly	~8000%	-20 °C	Almost 100%	Yes	This work

Note: “/” means not given.

Reference:

[S1] S. Sun, L.-B. Mao, Z. Lei, S.-H. Yu, H. Cölfen, *Angew. Chem. Int. Ed.* **2016**, *55*, 11765.

[S2] X. Huang, P. Wu, *Adv. Funct. Mater.* **2020**, *30*, 1910048.

[S3] L. L. da Luz, R. Milani, J. F. Felix, I. R. B. Ribeiro, M. Talhavini, B. A. D. Neto, J. Chojnacki, M. O. Rodrigues, S. A. Júnior, *ACS Appl. Mater. Interfaces* **2015**, *7*, 27115.

[S4] J. Huang, P. Wu, *Nano-Micro Lett.* **2020**, *13*, 15.

[S5]G. Ge, W. Yuan, W. Zhao, Y. Lu, Y. Zhang, W. Wang, P. Chen, W. Huang, W. Si, X. Dong, *J. Mater. Chem. A* **2019**, *7*, 5949.

[S6] H. Xu, Y. Tan, W. Rao, D. Wang, S. Xu, W. Liao, Y.-Z. Wang, *J. Colloid Interface Sci.* **2019**, *542*, 281.

[S7] H.-S. Yang, S. Cho, Y. Eom, S.-A. Park, S. Y. Hwang, H. Jeon, D. X. Oh, J. Park, *Macromol. Res.* **2021**, *29*, 140.

[S8] S.-H. Shin, Y. Eom, E. S. Lee, S. Y. Hwang, D. X. Oh, J. Park, *Adv. Healthcare Mater.* **2020**, *9*, 2000876.

[S9] M. Karolina Pierchala, F. B. Kadumudi, M. Mehrali, T.-G. Zsurzsan, P. J. Kempen, M. P. Serdeczny, J. Spangenberg, T. L. Andresen, A. Dolatshahi-Pirouz, *ACS Nano* **2021**, *15*, 9531.

[S10] C. Zhang, Y. Zhou, H. Han, H. Zheng, W. Xu, Z. Wang, *ACS Nano* **2021**, *15*, 1785.

[S11] S. Zhang, B. Xu, X. Lu, L. Wang, Y. Li, N. Ma, H. Wei, X. Zhang, G. Wang, *J. Mater. Chem. C* **2020**, *8*, 6763.

[S12] Z. Deng, T. Hu, Q. Lei, J. He, P. X. Ma, B. Guo, *ACS Appl. Mater. Interfaces* **2019**, *11*, 6796.

[S13] J. Wei, J. Xie, P. Zhang, Z. Zou, H. Ping, W. Wang, H. Xie, J. Z. Shen, L. Lei, Z. Fu, *ACS Appl. Mater. Interfaces* **2021**, *13*, 2952.

[S14] X. Li, L. He, Y. Li, M. Chao, M. Li, P. Wan, L. Zhang, *ACS Nano* **2021**, *15*, 7765.
